# Identification and functional analysis of a rare variant of gene *DHX37* in a patient with 46,XY disorders of sex development

**DOI:** 10.1002/mgg3.2453

**Published:** 2024-05-20

**Authors:** Wei Jiang, Jing Yu, Yu Mao, Yunman Tang, Li Cao, Qin Du, Jianan Li, Jiyun Yang

**Affiliations:** ^1^ Sichuan Provincial Key Laboratory for Human Disease Gene Study, Centre for Medical Genetics, Sichuan Academy of Medical Sciences, Sichuan Provincial People's Hospital University of Electronic Science and Technology of China Chengdu China; ^2^ Research Unit for Blindness Prevention of Chinese Academy of Medical Sciences (2019RU026), Sichuan Academy of Medical Sciences Chengdu China; ^3^ Meishan Women and Children's Hospital Alliance Hospital of West China Second University Hospital, Sichuan University Meishan China; ^4^ Department of Pediatric Surgery, Sichuan Provincial People's Hospital University of Electronic Science and Technology of China Chengdu China

**Keywords:** 46,XY DSD, *DHX37*, disorders of sex development, WES

## Abstract

**Background:**

46,XY sex reversal 11 (SRXY11) [OMIM#273250] is characterized by genital ambiguity that may range from mild male genital defects to gonadal sex reversal in severe cases. *DHX37* is an RNA helicase that has recently been reported as a cause of SRXY11. So far, a total of 21 variants in *DHX37* have been reported in 58 cases with 46,XY disorders of sex development (DSD).

**Methods:**

Whole exome sequencing (WES) was conducted to screen for variations in patients with 46,XY DSD. The subcellular localization of mutant DHX37 proteins was detected by immunofluorescence. And the levels of mutant DHX37 proteins were detected via Western blotting.

**Results:**

A novel pathogenic variant of *DHX37* was identified in a patient with 46,XY DSD c.2012G > C (p.Arg671Thr). Bioinformatics analysis showed that the protein function of the variant was impaired. Compared with the structure of the wild‐type DHX37 protein, the number of hydrogen bonds and interacting amino acids of the variant protein were changed to varying degrees. In vitro assays revealed that the variant had no significant effect on the intracellular localization of the protein but significantly reduced the expression level of the protein.

**Conclusions:**

Our finding further expands the spectrum of the *DHX37* variant and could assist in the molecular diagnosis of 46,XY DSD patients.

## Graphical abstract

46,XY sex reversal 11 (SRXY11) [OMIM#273250] is characterized by genital ambiguity that may range from mild male genital defects to gonadal sex reversal in severe cases. DHX37 is an RNA helicase that has recently been reported as a cause of SRXY11. So far, a total of 21 pathogenic variants in DHX37 have been reported in 58 cases with 46,XY disorders of sex development (DSD).Our finding further expands the spectrum of the DHX37 variant and could assist in the molecular diagnosis of 46,XY DSD patients.

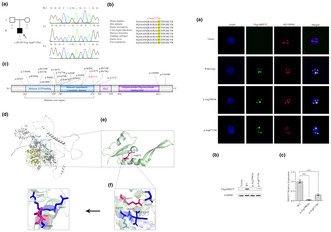

## 1 INTRODUCTION

Disorders of sex development (DSDs) are congenital birth defects that occur in one out of every 4500–5000 live births (Hughes et al., 2007; Lee et al., 2016). They are classified according to sex chromosomes into 46,XY DSD and 46,XX DSD (Houk et al., 2006; Hughes et al., 2006). Among DSD patients, 46,XY is the most common type, occurring in 75% of individuals, while 46,XX DSD affects approximately 10%–15%. The remainder have structural or numerical abnormalities in their sex chromosomes (Rodie et al., 2011). The etiology of DSD includes genetic factors, environmental factors, and combinations of the two (Arboleda et al., 2014; Bouty et al., 2015; Dave et al., 2019). To date, over 40 distinct phenotypes and more than 60 genes associated with DSD have been reported, indicating a significant clinical and genetic heterogeneity of the disease (Parivesh et al., 2019; Tenenbaum‐Rakover et al., 2021). Although variations in multiple genes, including *SRY*, *AR*, *NR5A1*(SF1), *SOX9, CYP21A2*, and *FOXL2*, have been determined to be involved in DSD, the genetic causes of 60%–80% of cases are still elusive (Baxter et al., 2015; Dong et al., 2016; Eggers et al., 2016; Fan et al., 2017; Hughes et al., 2019; Kim et al., 2017; Ozen et al., 2017; Wang et al., 2018; Xu et al., 2019).

Asp‐Glu‐Ala‐His‐box helicase 37 (DHX37‐NM_032656.4) is a member of DEAH‐box helicase family 2, which is located on chromosome 12q24.31 and encodes an RNA helicase protein. It contains four domains: a helicase ATP‐binding domain (RecA1), a helicase superfamily c‐terminal domain (RecA2), a helicase‐associated domain (HA2), and an oligonucleotide/oligosaccharide‐binding fold domain (da Silva et al., 2019). In 2019, Da Silva et al. first reported the association of a heterozygous mutation of the *DHX37* gene with DSD phenotypes. Affected individuals with the 46,XY karyotype exhibited a variety of genital abnormalities, ranging from hypospadias, undescended testicles, severe hypospadias, and ambiguous genitalia to normal‐appearing female external genitalia. So far, 21 pathogenic variants of the *DHX37* gene have been identified in 58 DSD pedigrees or sporadic patients (Buonocore et al., 2019; da Silva et al., 2019; McElreavey et al., 2019; Shaomei et al., 2022; Zidoune et al., 2021). Most pathogenic variants of the *DHX37* gene show an autosomal dominant inheritance pattern. Some have variable expressivity, a sex‐ limited phenotype, or reduced penetrance (da Silva et al., 2019; Zidoune et al., 2021).

In the current investigation, we described a Chinese patient with a 46,XY DSD caused by a novel heterozygous variant of the *DHX37* gene: c.2012G > C (p.Arg671Thr). Additionally, we explore the effect of this variation on protein expression and localization, which expands the mutation spectrum of*DHX37*.

## 2 MATERIALS AND METHODS

### 2.1 Subject

This study was approved by the Institutional Review Board of Sichuan Provincial People's Hospital. Written informed consent was signed by the patient and his parents. And the patient was thoroughly clinically evaluated, including through a genital examination of gonads, chromosome karyotype testing, and hormone testing. The case was eventually diagnosed as 46,XY DSD.

### 2.2 Whole exome sequencing

Genomic DNA was extracted from the whole blood of all family members of the individual with the DSD using a GentraPuregen kit (Qiagen) according to the manufacturer's protocol. Exome capture was performed using the Agilent SureSelectXT Human All Exon Kit V6r2 (Agilent). Libraries were sequenced as 150‐bp paired end reads on an Illumina HiSeq 2000 instrument. These reads were aligned with the Human Genome Reference Database (GRCh38/hg38) using the BWA MEM algorithm. Single‐nucleotide variants and small insertions and deletions were detected with the GATK HaplotypeCaller. Copy number variants were recomputed with the EXCAVATOR2 software.

Annotation and pathogenicity analyses of variants were performed as previously described (Ludwig et al., 2014). In brief, sequence variations were compared with the 1000 Genomes Project (http://www.1000genomes.org/), the GnomAD (https://gnomad.broadinstitute.org/), and an internal whole exome sequencing (WES) database of 2114 Han Chinese records. Multiple tools were used for in silico analysis, including SIFT (http://sift.jcvi.org/), PROVEAN (http://provean.jcvi.org/), Polyphen‐2 (http://genetics.bwh.harvard.edu/pph2/), Mutation Taster (http://www.mutationtaster.org), and CADD (https://cadd.gs.washington.edu/snv). Variants were categorized as benign, likely benign with uncertain significance, likely pathogenic, or pathogenic according to the interpretation guidelines of the American College of Medical Genetics and Genomics. We gave priority to 165 genes that cause DSD (Table S1).

### 2.3 Validation of variants and inheritance analysis

Sanger sequencing was used to confirm the candidate pathogenic variant detected using WES. Specific primers (F: 5′‐GACTCGGTTGTGTGTTGTG‐3′ and R: 5′‐AGCATCCTTCCCTTGTTCCT‐3′) were used to amplify the region of interest. The PCR products were subsequently visualized using a 1% agarose gel, and bidirectional sequencing was carried out using an ABI PRISM3130xl Genetic Analyzer (Thermo Fisher Scientific, Inc.).

### 2.4 In silico analysis of variants of *DHX37*

Amino acid sequence homology analysis was performed using HomoloGene (https://www.ncbi.nlm.nih.gov/homologene). The wild‐type and mutant 3D structures of the DXH37 protein were modeled using the online protein model prediction server Alphafold (https://alphafold.ebi.ac.uk/). Simultaneously, the protein structure changes and amino acid interactions were analyzed using PyMOL (https://pymol.org/2/).

### 2.5 Plasmid construction

A pcDNA3.1‐Flag‐C‐DHX37 (NM_032656.4) plasmid was obtained from YouBio. The p.Arg308Gln and p.Arg671Thr mutations were introduced into the pcDNA3.1‐Flag‐C‐*DHX37* (NM_032656.4) plasmid using a Q5® Site‐Directed Mutagenesis Kit (New England Biolabs, USA), generating pcDNA3.1‐Arg308Gln and pcDNA3.1‐Arg671Thr. The p.Arg308Gln mutation of *DHX37* was the hotspot for the *DHX37* variant, as it was detected in about 38% of the cases (Buonocore et al., 2019; da Silva et al., 2019; de Oliveira et al., 2023; McElreavey et al., 2019; Zhang et al., 2023; Zidoune et al., 2021). Thus, pcDNA3.1‐Arg308Gln was chosen as a reference plasmid. These plasmids were thus constructed and confirmed via Sanger sequencing.

### 2.6 Immunofluorescence

The empty vector (pcDNA3.1‐Flag‐C), wild‐type vector (pcDNA3.1‐Flag‐C‐DHX37), and mutant vector were transfected into COS7 cells in 24‐well plates. Protein localization was detected using immunocytochemistry 48 h after transfection. The localization of wild‐type and mutant DHX37 was detected using anti‐FLAG (#14793, Cell Signaling Technology). B23/NPM1 (#60096‐1‐Ig, Proteintech) was used to label nucleoli. Fluorescence was visualized using a Zeiss LSM 800 confocal microscope (Zeiss, Oberkochen, Germany).

### 2.7 Protein expression analysis

In this step, 80 ng of pcDNA3.1‐EGFP plasmid and 100 ng of each empty vector (pcDNA3.1‐Flag‐C), wild‐type vector (pcDNA3.1‐Flag‐C‐DHX37), and mutant vectors (p.Arg308Gln and p.Arg671Thr) were transfected into HEK293T cells in six‐well plates. After 48 h of transfection, fluorescent protein could be detected in the cells under a fluorescence microscope. The protein extracted from the cells indicated that the transfection efficiency of the three vectors was 60%–70%. GAPDH was used as an internal control. Anti‐FLAG (#14793, Cell Signaling Technology) was used to detect the DHX37 protein, and anti‐GAPDH (#SA00001‐2, Proteintech) was used as a loading control. The intensity of the protein bands was analyzed using Image J software (National Institutes of Health). Each experiment was conducted three times, independently.

## 3 RESULTS

### 3.1 A variant of *DHX37* identified in a patient with DSD

**FIGURE 1:**
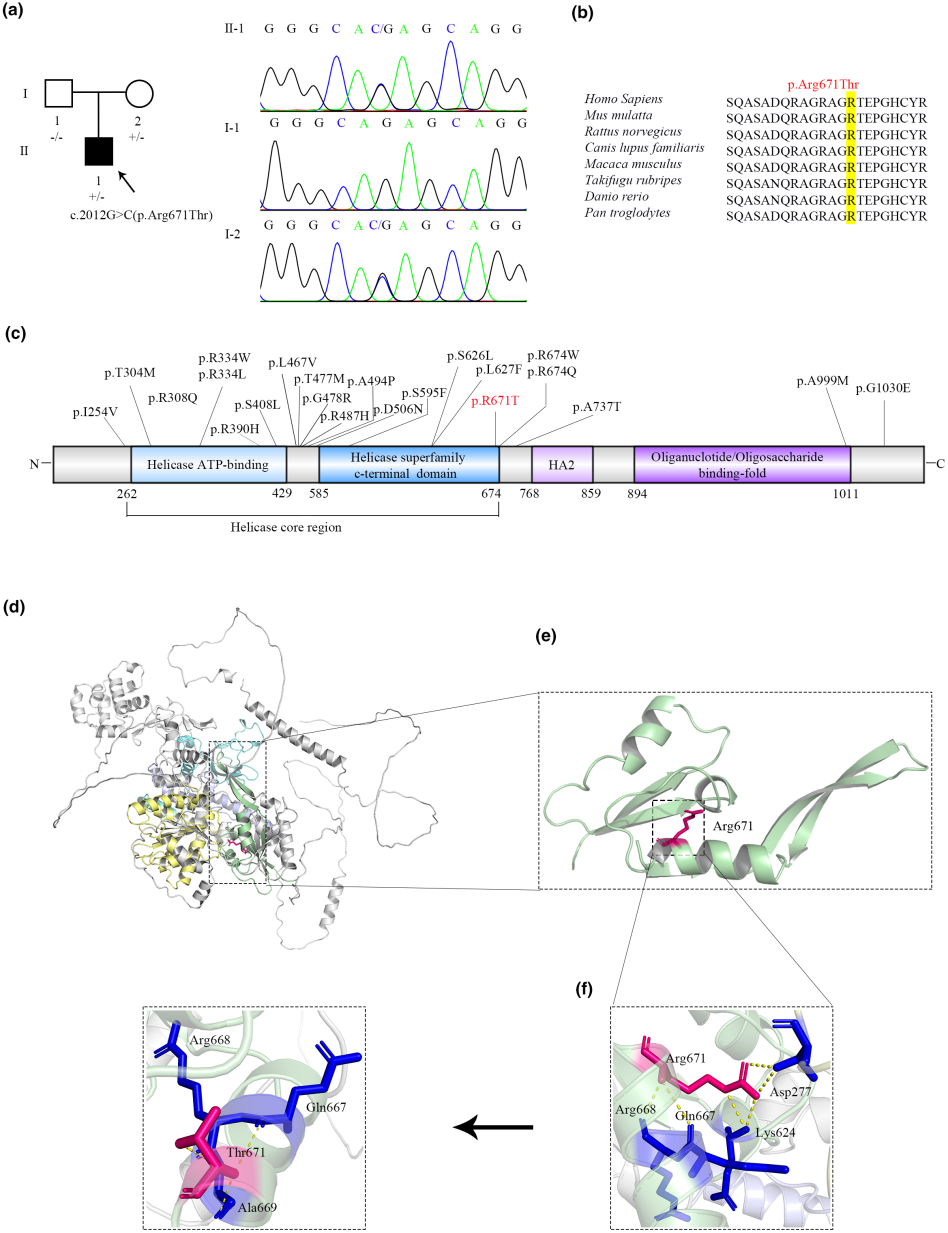
Pedigree and in silico analysis of a novel DHX37 variant. (a) A family with *DHX37* variation c.2012G > C (p.Arg671Thr). Each pedigree shows affected (dark symbols) and unaffected (open symbols) individuals, with squares representing males and circles representing females. The probands are indicated with oblique arrows; −/− represents the wild‐type allele of the homozygous state, and +/− stands for that of the heterozygous state. (b) The evolutionary conservation of two amino acid variants of the DHX37 protein among different species. Mutated amino acids are indicated by yellow shading. (c) Schematic of the DHX37 protein labeled with previously reported and newly identified *DHX37* variants, including conserved motifs of the helicase core region (helicase ATP‐binding and helicase superfamily c‐terminal domain), helicase‐associated domain (HA2), and oligonucleotide/oligosaccharide‐binding fold. (d) Crystal structure of the DHX37 protein. The four functional domains are shown in yellow, green, mauve, and light blue, respectively. (e) Close‐up view of the helicase superfamily c‐terminal domain (RecA2). (f) Close‐up view of the interaction between residue 671 and other residues.

The patient was a 14‐year‐old male with pubertal retardation and partial gonadal dysgenesis. A physical examination and ultrasound revealed ambiguous external genitalia, including perineal hypospadias, micropenis (53.0 × 10.0 mm), and bilateral inguinal cryptorchidism. The ultrasound showed that the uterus and ovary were not visible within the pelvic cavity, the scrotum was empty on both sides, and a testicular echo was detected in the inguinal canal, which measured around 19.0 × 7.0 × 7.6 mm on the right side and 19.0 × 9.0 × 16.0 mm on the left side. The results of the hormonal tests were as follows: The hormonal tests showed that testosterone was <0.45 ng/mL, androstenedione was <0.30 ng/mL, AMH was <0.06 ng/mL, and InhB was <10 pg/mL, while there was 23.34 mIU/mL of LH and 74.92 mIU/mL of FSH. He was eventually diagnosed with a 46,XY DSD. In addition, the heterozygous missense variant c.2012G > C (p. Arg671Thr) in *DHX37* was confirmed in the affected individual through WES methods. Sanger sequencing showed that it was inherited from the unaffected mother (Figure 1a). The clinical features of previously reported patients with *DHX37* variants are shown in Supplementary Table S2.

### 3.2 In silico analysis of the *DHX37* variant

Multiple amino acid sequence alignment of *DHX37* showed that the mutant residue sites were evolutionarily conserved in eight species (Figure 1b). To predict the molecular consequences of the variant, the alpha fold was used to predict the crystal structure model of DHX37 (Figure 1d). The model of the missense variant was refined using PyMOL.

c.2012G>C (p.Arg671Thr) was located in the helicase superfamily c‐terminal domain of the helicase core region in DHX37 (Figure 1c,e). The PyMOL analysis demonstrated that wild‐type Arg671 interacted with 624Lys, 667Gln, and 668Arg in the same domain and 277Asp in the helicase ATP‐binding domain. However, when Thr was substituted for Arg, it interacted with 667Gln, 668Arg, and 669Ala residues (Figure 1f).

The predictive functional effects and population distribution frequencies of previously reported and newly identified *DHX37* variants are summarized in Table 1.

**TABLE 1:** Bioinformatics analysis of all identified *DHX37* gene variants associated with 46, XYDSD.

ID	Variation	gnomAD	SIFT	PROVEAN	Polyphen‐2	MutationTaster	Pathogenicity classification	CADD
1	c.760A>G(p.I254V)	0.00002909	Tolerated (0.58)	Neutral (−0.598)	Benign (0.112)	Disease causing	VUS	23.3
2	c.911C>T(p.T304M)	0.0001995	Damaging (0)	Deleterious (−5.957)	Probably damaging (1)	Disease causing	Pathogenic	26.1
3	c.923G>A(p.R308Q)	0.00003187	Damaging (0)	Deleterious (−3.971)	Probably damaging (1)	Disease causing	Pathogenic	32
4	c.1000C>T(p.R334W)	0	Damaging (0)	Deleterious (−7.987)	Probably damaging (1)	Disease causing	Likely pathogenic	32
5	c.1001G>T(p.R334L)	0	Damaging (0)	Deleterious (−6.989)	Probably damaging (1)	Disease causing	Likely pathogenic	29
6	c.1169G>A(p.R390H)	0.00000399	Damaging (0)	Deleterious (−4.964)	Probably damaging (1)	Disease causing	VUS	31
7	c.1223C>T (p.S408L)	0.00001064	Damaging (0)	Deleterious (−5.824)	Probably damaging (0.999)	Disease causing	VUS	50
8	c.1399C>G(p.L467V)	0.000003999	Damaging (0)	Deleterious (−2.967)	Probably damaging (0.999)	Disease causing	VUS	22.9
9	c.1430C>T(p.T477M)	0.00001065	Damaging (0.02)	Deleterious (−4.964)	Probably damaging (1)	Disease causing	Likely pathogenic	28
10	c.1432G>A(p.G478R)	0	Damaging (0)	Deleterious (−7.867)	Probably damaging (1)	Disease causing	VUS	28.8
11	c.1460G>A(p.R487H)	0.00003595	Damaging (0.015)	Deleterious (−4.546)	Benign (0.227)	Disease causing	Likely benign	24.2
12	c.1474G>C(p.A492P)	0	Damaging (0)	Deleterious (−3.694)	Benign (0.317)	Disease causing	VUS	24.7
13	c.1516G>A(p.D506N)	0.00007960	Damaging (0)	Neutral (−0.854)	Benign (0.176)	Disease causing	Benign	18.45
14	c.1784C>T(p.S595F)	0	Damaging (0.001)	Deleterious (−5.568)	Benign (0.331)	Disease causing	Pathogenic	23.4
15	c.1877C>T(p.S626L)	0	Damaging (0.002)	Deleterious (−5.624)	Probably damaging (1)	Disease causing	Likely pathogenic	25.9
16	c.1879C>A (p.L627F)	0	Damaging (0.04)	Deleterious (−3.794)	Probably damaging (1)	Disease causing	VUS	25.2
**17**	**c.2012G>C(p.R671T)**	**0**	**Damaging (0.004)**	**Deleterious (−5.657)**	**Probably damaging (1)**	**Disease causing**	**VUS**	25.6
18	c.2021G>A(p.R674Q)	0	Damaging (0.001)	Deleterious (−3.794)	Probably damaging (1)	Disease causing	Pathogenic	29.9
19	c.2020C>T(p.R674W)	0	Damaging(0.001)	Deleterious (−7.421)	Probably damaging (1)	Disease causing	Likely pathogenic	27.8
20	c.2209G>A(p.A737T)	0.00008273	Damaging (0)	Deleterious (−3.560)	Probably damaging (1)	Disease causing	VUS	24.8
21	c.2995G>A(p.V999M)	0.0003170	Damaging (0)	Neutral (−2.497)	Probably damaging (0.998)	Disease causing	Likely benign	24.5
22	c.3089G>A(p.G1030E)	0.00003037	Damaging (0.005)	Deleterious (−6.754)	Probably damaging (1)	Disease causing	VUS	24.1

*Note*: *DHX37*: NM_032656. Output predictions of each tool—SIFT classification: damaging, tolerated; PROVEAN classification: Deleterious, Neutral; PolyPhen‐2 classification: probably damaging, benign; Mutation Taster classification: Disease‐causing automatic, disease‐causing, polymorphism. Polymorphism automatic; Pathogenic analysis classification: According to the ACMG, pathogenic, likely pathogenic, benign, likely benign, variant of uncertain significance (VUS). CADD: The top 10% of the Reference genome single‐nucleotide variants for CADD score were 10, the top 1% were 20, and the top 0.1% were 30, and so on. Bolded text represents newly identified variant in this study.

### 3.3 Effect of *DHX37* gene variants on protein localization

**FIGURE 2:**
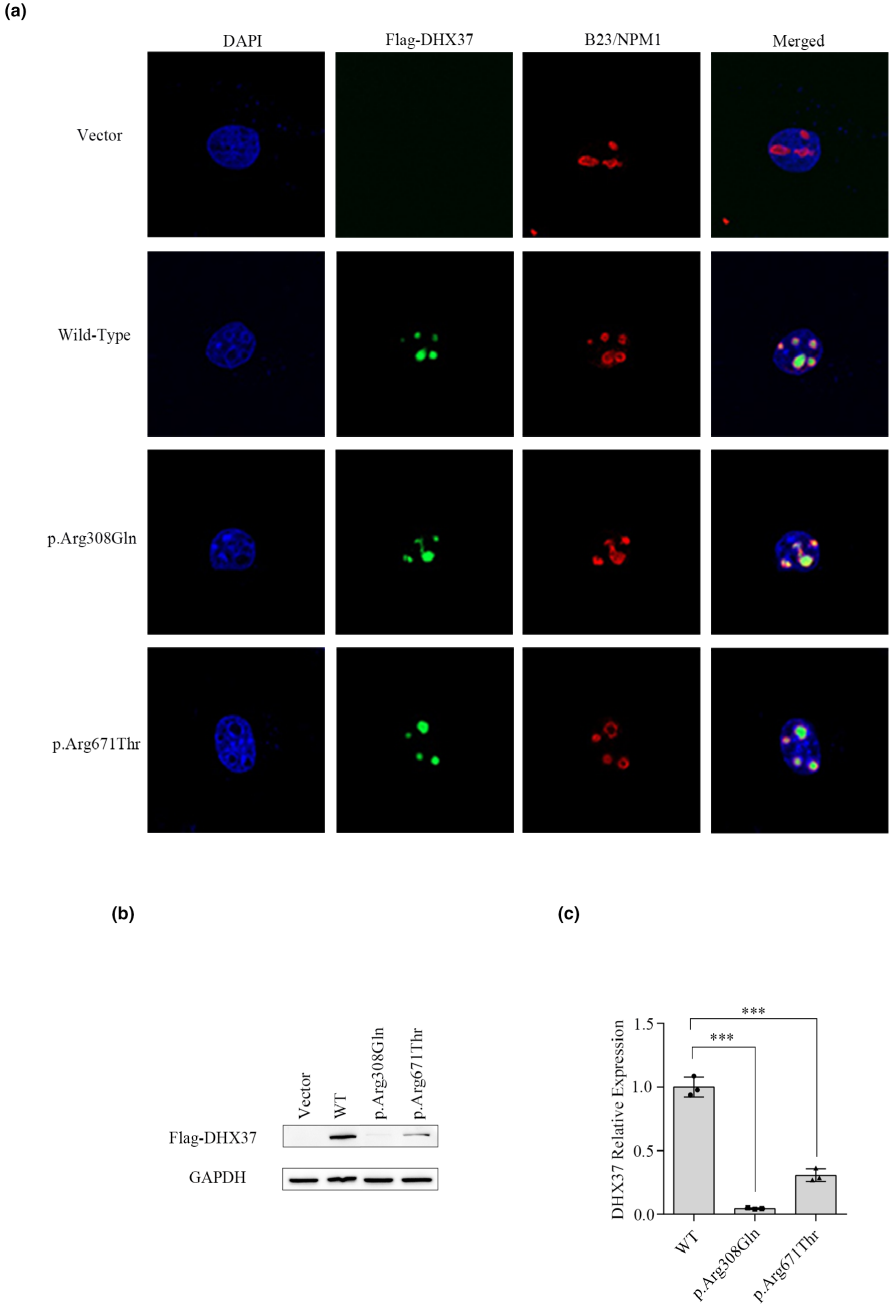
Subcellular localization and expression analysis of two mutant DHX37 proteins. (a) Subcellular localization of two mutant DHX37 proteins. COS7 cells were transfected with flag‐tagged WT‐DHX37 or the DHX37 variant. The subcellular localization of DHX37 proteins was detected using anti‐FLAG (green). Highly conserved nucleolar proteins are stained with B23/NPM1 (red). The nucleus is stained with DAPI (blue). The subcellular localization of mutant DHX37 protein was similar to that of the wild‐type, as it was located in the nucleoli. (b) Expression of DHX37 protein as determined by western blot assay in 293 T cells transfected with wild‐type and mutant*DHX37*plasmids. (c) Quantitation of the western blot results. The relative ratios of proteins were presented as mean ± SEM. Each experiment was conducted three times, independently. Compared with the cells transfected with pcDNA3.1‐WT, those with pcDNA3.1‐Arg308Gln and pcDNA3.1‐Arg671Thr displayed decreased DHX37 expression (*p*< 0.01). *** representing *p*< 0.001.

To determine the subcellular localization of the mutant DHX37 protein, we performed immunofluorescence analysis. COS7 cells were transiently transfected with wild‐type or mutant *DHX37* plasmids (p.Arg671Thr and p.Arg308Gln). The c.923G > A (p.Arg308Gln) variant of the *DHX37* gene was a common pathogenic variant, so this variant served as the reference (Buonocore et al., 2019; da Silva et al., 2019; de Oliveira et al., 2023; McElreavey et al., 2019; Zhang et al., 2023; Zidoune et al., 2021). The results showed that the wild‐type and mutant proteins were predominantly localized in the nucleolei of COS7 cells (Figure 2a). There were no significant differences between the localization of the mutant DHX37 protein and that of the wild‐type protein.

### 3.4 Effect of *DHX37* gene variants on protein expression

To detect the effects of the missense variant on protein expression, we transfected 293 T cells with wild‐type and mutant DHX37 plasmids. The levels of DHX37 protein were detected via western blotting. As shown in Figure2b,c, compared with the cells transfected with pcDNA3.1‐WT, those with pcDNA3.1‐Arg308Gln and pcDNA3.1‐Arg671Thr displayed a lower level of DHX37 (*p*< 0.01). The results indicated that mutant *DHX37* decreased the protein levels of DHX37.

## 4 DISCUSSION

DSD is a rare and clinically heterogeneous disease. In recent years, with the application of high‐throughput sequencing technology to the field of genetic disorders, an increasing number of genes that cause DSD have been identified (Audi et al., 2018). In this study, we used whole‐exome sequencing to detect the genetic causes of a case of DSD. The results revealed a missense variant in the *DHX37* gene: c.2012G > C (p.Arg671Thr). Variations of *DHX37* are reported to lead to neurodevelopmental disorders and 46,XY DSD (Buonocore et al., 2019; Karaca et al., 2015; McElreavey et al., 2019; Paine et al., 2019; Zidoune et al., 2021). To date, 21 heterozygous variants of*DHX37*have been identified in 58 patients with 46,XY DSD (Buonocore et al., 2019; da Silva et al., 2019; de Oliveira et al., 2023; Globa et al., 2022; Gomes et al., 2022; Kulkarni et al., 2023; McElreavey et al., 2019; Shaomei et al., 2022; Wan et al., 2023; Yang et al., 2023; Zhang et al., 2023; Zidoune et al., 2021). All affected individuals have manifestations of gonadal dysgenesis, varying from micropenis and bilateral rudimentary gonadal tissue to absent, ambiguous, or atypical genitalia. The clinical features of previously reported patients with *DHX37* variants are shown in Table S2. Of the 58 cases, variants in 9 cases were de novo, variants in 16 cases were inherited from the mother, and variants in 28 cases were of unknown origin. One case carried homozygous variants that showed an autosomal recessive mode of inheritance. 46,XX individuals carrying the *DHX37* mutation were clinically asymptomatic, whereas 46,XY individuals have varying degrees of clinical symptoms, suggesting that DSD patients associated with *DHX37* variants have variable expressivity and a sex‐limited phenotype. It is worth noting that four *DHX37* variants were inherited from fathers with completely normal phenotypes, suggesting reduced penetrance (Buonocore et al., 2019; da Silva et al., 2019; McElreavey et al., 2019; Zidoune et al., 2021). In our study, the variant was maternal. Similarly, the clinical characteristics of the patient were consistent with previous studies (da Silva et al., 2019; McElreavey et al., 2019; Zidoune et al., 2021).

The *DHX37* variations for 46,XY DSD are mainly mapped to the helicase core domain, according to previous reports. In our study, the p.Arg671Thr variant was located in the helicase superfamily c‐terminal domain of the *DHX37* helicase core domain. The p.Arg671Thr mutation leads to a substitution of arginine for threonine at position 671. The wild‐type residue charge was positive, and the mutant residue charge was neutral. The substitution of amino acids results in more hydrophobic residue and a loss of hydrogen bonds. The change in charge probably disturbed the correct protein folding and catalysis of the RNA helicase. To assess the pathogenic potential of the *DHX37* variant, the subcellular localization of the variant was detected through immunofluorescence. No difference in localization was observed. Both the wild‐type and mutant proteins were primarily localized in the nucleus. However, western blot analyses showed that the variant severely reduced the expression levels of the mutant protein. According to the ACMG guidelines, p.Arg671Thr is regarded as pathogenic. However, the molecular mechanism by which variants in *DHX37* lead to DSD remains unexplained.

*DHX37* has also been reported to participate in many RNA‐related processes, such as ribosome biogenesis, transcription, splicing, translation, and degradation (Bleichert & Baserga,^2007^; Jankowsky et al., 2001). RNA helicases are particularly important in regulating ribosomal subunit assembly and are involved in numerous structural transitions and complex formations (Russon et al., 2021). DHX37 is required for pre‐40s maturation, and its absence impairs various steps in the maturation process of 18S rRNA, thereby reducing the levels of both the mature 18S rRNA and 40S subunit (Choudhury et al., 2019). Due to the critical role of ribosomes, the disturbance of human ribosome production is associated with genetic diseases known as ribosomopathies (McElreavey et al., 2022; Mills & Green,^2017^). There have been reports that homozygous, compound heterozygous, and de novo heterozygous missense variants in *DHX37* are associated with neurodevelopmental disorder with brain anomalies and with or without vertebral or cardiac anomalies (NEDBAVC; OMIM# 618731) with an apparent absence of DSD (Karaca et al., 2015; Paine et al., 2019). Although ribosomes are present in all types of cells, variations in ribosomal proteins or assembly factors produce tissue‐specific phenotypes and diseases (Arboleda et al., 2014; Draptchinskaia et al., 1999). Defects in ribosome homeostasis may affect the synthesis of specific differentiation or proliferation regulators, which may explain this phenotypic difference (Ludwig et al., 2014). McElreavey et al. have proposed a link between ribosome biogenesis and tissue‐specific gene expression profiles (McElreavey et al., 2022). However, it still doesn't explain the phenomenon in which various pathogenic variants in *DHX37* result in DSD or NEDBAVC.

In summary, we have reported a novel variant of the *DHX37* gene in connection with the clinical characteristics of a Chinese patient with 46,XY DSD. In vitro assays revealed that the variant of DHX37 had no significant effect on the intracellular localization of protein but significantly reduced the expression levels of protein. The finding of a novel variant further expands the existing spectrum of *DHX37* variants and assists in the molecular diagnosis of 46,XY DSD patients. However, due to the limited number of *DHX37* variants in cases of DSD and NEDBAVC, it is currently difficult to establish a clear phenotype–genotype correlation. This correlation needs to be validated in large population‐based studies, and further experiments are needed to elucidate the pathogenic mechanism by which *DHX37* leads to 46,XY DSD and NEDBAVC.

## AUTHOR CONTRIBUTIONS

Jiyun Yang conceived and designed the experiments. Wei Jiang and Jing Yu performed the experiments. Jiyun Yang and Yu Mao analyzed the data. Yu Mao, Yunman Tang, Qin Du, Li Cao, and Jianan Li collected samples. Jiyun Yang and Yu Mao drafted the manuscript. Jiyun Yang revised the manuscript and gave final approval of the version to be published.

## CONFLICT OF INTEREST STATEMENT

All authors declare no competing interests.

## ETHICS STATEMENT

Written informed consent was signed by the patient and his parents. This study was approved by the Institutional Review Board of Sichuan Provincial People’s Hospital.

## Supporting information


Table S1.

Table S2.

## Data Availability

All data generated or analyzed during this study are included in this published article or the data repositories listed in the references.
