# Supplementary material for: Identification and functional analysis of a rare variant of gene DHX37 in a patient with 46,XY disorders of sex development
Source: Mol Genet Genomic Med. 2024 May 20;12(5):e2453. doi: 10.1002/mgg3.2453 (PMC11106588; doi:10.1002/mgg3.2453)
Supplement: Supplementary file 1 — Table S1. [file MGG3-12-e2453-s001.docx]

Supplementary Table 1 165 disease-causing genes for DSD

| *A2ML1* | *BMP15* | *DHH* | *GK2* | KISS1 | NR5A1 | *RIT1* | *STAR* |
| --- | --- | --- | --- | --- | --- | --- | --- |
| *AARS2* | *BRAF* | *DHX37* | *GNAS* | *KISS1R* | *NRAS* | *RNF216* | *SYCP3* |
| *AKR1C2* | *CATSPER1* | *DIAPH2* | *GNRH1* | *KLHL10* | *NSDHL* | *ROR2* | *TAC3* |
| *AKR1C4* | *CBX2* | *DMRT1* | *GNRHR* | *KMT2D* | *NSMF* | *RSPO1* | *TACR3* |
| *AMH* | *CCDC28B* | *DPY19L2* | *GOPC* | *KRAS* | *ORC1* | *RXFP2* | *TAF4B* |
| *AMHR2* | *CD96* | *DUSP6* | *HARS2* | *LARS2* | *PHF6* | *RXRA* | *TMEM67* |
| *ANOS1* | *CDKN1C* | *ERCC6* | *HDAC8* | *LHB* | *POF1B* | *RXRB* | *TRIM32* |
| *AR* | *CEP19* | *ERCC8* | *HFE* | *LHCGR* | *POLR3A* | *SDCCAG8* | *TSPYL1* |
| *ARL6* | *CEP290* | *ESR1* | *HFM1* | *LZTFL1* | *POLR3B* | *SEMA3A* | *TTC8* |
| *ARX* | *CFTR* | *FEZF1* | *HGF* | *MAMLD1* | *POMC* | *SEMA3E* | *TWNK* |
| *ATM* | *CHD7* | *FGD1* | *HOXA13* | *MAP3K1* | *POR* | *SHOC2* | *USP9Y* |
| *ATRX* | *CHRM3* | *FGF17* | *HS6ST1* | *MCM9* | *PROK2* | *SLC26A8* | *WDPCP* |
| *AURKC* | *CLPP* | *FGF8* | *HSD17B3* | *MED12* | *PROKR2* | *SOS1* | *WDR11* |
| *BBS1* | *CYB5A* | *FGFR1* | *HSD17B4* | *MID1* | *PSMC3IP* | *SOX10* | *WNT3* |
| *BBS10* | *CYP11B1* | *FIGLA* | *HSD3B2* | *MKKS* | *PTPN11* | *SOX3* | *WNT4* |
| *BBS12* | *CYP17A1* | *FLRT3* | *ICK* | *MKRN3* | *RAB23* | *SOX9* | *WNT5A* |
| *BBS2* | *CYP19A1* | *FMR1* | *IGSF10* | *MKS1* | *RAB3GAP2* | *SPATA16* | *WT1* |
| *BBS4* | *CYP21A2* | *FOXL2* | *IL17RD* | *NAA10* | *RAF1* | *SPRY4* | *ZMYND15* |
| *BBS5* | *DAZL* | *FSHR* | *INSL3* | *NANOS1* | *RASA2* | *SRD5A2* |  |
| *BBS7* | *DCAF17* | *GATA4* | *IRF6* | *NOBOX* | *REN* | *SRY* |  |
| *BBS9* | *DHCR7* | *GK* | *KDM6A* | *NR0B1* | *RIPK4* | *STAR* |  |
