# Supplementary material for: Identification and functional analysis of a rare variant of gene DHX37 in a patient with 46,XY disorders of sex development
Source: Mol Genet Genomic Med. 2024 May 20;12(5):e2453. doi: 10.1002/mgg3.2453 (PMC11106588; doi:10.1002/mgg3.2453)
Supplement: Supplementary file 2 — Table S2. [file MGG3-12-e2453-s002.docx]

Supplementary Table2 Clinical features of previous reported patients with *DHX37* variants

| **Variable** | Patient 1 | Patient 2 | Patient 3 | Patient 4 | Patient 5 | Patient 6 | Patient 7 | Patient 8 | Patient 9 | Patient 10 | Patient 11 |
| --- | --- | --- | --- | --- | --- | --- | --- | --- | --- | --- | --- |
| Sex of rearing | Male | Male | Male | Female | Male | Male | Male | Male | Male | Female | Male |
| Karyotype | 46,XY | 46,XY | 46,XY | 46,XY | 46,XY | 46,XY | 46,XY | 46,XY | 46,XY | 46,XY | 46,XY |
| Age at presentation | 2y2m | 1y10m | 14y | 1y10m | 7m2d | 24d | Birth | 4y | 2y | 18y | 10y |
| Diagnosis | ETRS | ETRS | ETRS | ETRS | ETRS | ETRS | ETRS | ETRS | PGD | PGD | ETRS |
| External genitalia | Micropenis | Micropenis | Micropenis | Micropenis | Micropenis | Micropenis | Micropenis | Micropenis | Micropenis | Atypical | Micropenis |
| Internal genitalia | Wolffian  structures and Müllerian structures present | no Müllerian structures, Wolffian structures present | Wolffian  structures and Müllerian structures present | Wolffian  structures and Müllerian structures present | no Müllerian structures, Wolffian structures present | Wolffian  structures and Müllerian structures present | Wolffian  structures and Müllerian structures present | Wolffian  structures and Müllerian structures present | no Müllerian structures, Wolffian structures present | no Müllerian structures, Wolffian structures present | no Müllerian structures, Wolffian structures present |
| Gonad histology | Small  bilateral dysgenetic gonads | Left gonad not found; right dysgenetic gonad | No gonadal tissue found | Left gonad not found; small right dysgenetic gonad | Small bilateral dysgenetic gonads | Small bilateral dysgenetic gonads | No gonadal tissue | No gonadal tissue | Right gonad not found; left dysgenetic testis a with GCNIS | Bilateral dysgenetic gonads | No gonadal tissue |
| **LH, UI/L** |  |  |  |  |  |  |  |  |  |  |  |
| Value | 14.5 | 12 | 3.5 | 1.9 | <0.5 | <0.5 | <0.5 | <0.5 | 26 | NA | 19 |
| Ref. Range | 0.22–1.92 | 0.22–1.92 |  |  | 1.1–25 | 0.9–3 | <2 | 0.03–0.55 | 0.22–1.92 |  | l.2–7.8 |
| **FSH, UI/L** |  |  |  |  |  |  |  |  |  |  |  |
| Value | 117 | 133 | 87 | 56 | 10.9 | 9.5 | NA | 9 | 112 | NA | 43 |
| Ref. Range | 0.7–3.39 | 0.7–3.39 |  |  | 1.5–11.8 | <3 |  | 0.7–3.39 | 0.7–3.39 |  | 3.03-8.08 |
| **T, ng/mL** |  |  |  |  |  |  |  |  |  |  |  |
| Value | <10 | <10 | <10 | NA | 16 | <10 | 38 | 27 | 16 | NA | 21 |
| Ref. Range | 0.02–0.23 | 0.02–0.23 |  |  | 2.2–10.5 | 1.0–3.0 | 0.4–3 | 0.02–0.23 | 0.02–0.23 |  | 0-8.8 |
| **AMH, ng/mL** |  |  |  |  |  |  |  |  |  |  |  |
| Value | NA | NA | NA | NA | NA | NA | NA | NA | NA | NA | NA |
| Ref. Range |  |  |  |  |  |  |  |  |  |  |  |
| Ancestry | Brazilian | Brazilian | Brazilian | Brazilian | Chilean | Chilean | Argentinian | Argentinian | Argentinian | Brazilian | Brazilian |
| *DHX37*pathogenic variant,zygosity and inheritance | c.923G>A p.Arg308Gln heterozygous paternal | c.923G>A p.Arg308Gln heterozygous paternal | c.923G>A p.Arg308Gln heterozygous unknown | c.923G>A p.Arg308Gln heterozygous maternal | c.2020C>T p.Arg674Trp heterozygous maternal | c.2020C>T p.Arg674Trp heterozygous maternal | c.2020C>T p.Arg674Trp heterozygous maternal | c.2020C>T p.Arg674Trp heterozygous maternal | c.2020C>T p.Arg674Trp heterozygous maternal | c.1784C>T  p.Ser595Phe heterozygous maternal | c.1784C>T  p.Ser595Phe heterozygous maternal |
| reference | da Silva et al.2019 | da Silva et al.2019 | da Silva et al.2019 | da Silva et al.2019 | da Silva et al.2019 | da Silva et al.2019 | da Silva et al.2019 | da Silva et al.2019 | da Silva et al.2019 | da Silva et al.2019 | da Silva et al.2019 |

| **Variable** | Patient 12 | Patient 13 | Patient 14 | Patient 15 | Patient 16 | Patient 17 | Patient 18 | Patient 19 | Patient 20 | Patient 21 | Patient 22 |
| --- | --- | --- | --- | --- | --- | --- | --- | --- | --- | --- | --- |
| Sex of rearing | Male | Male to Female | Female | Female | Male to female | Female | Female | Female | Female | Female | Female |
| Karyotype | 46,XY | 46,XY | 46,XY | 46,XY | 46,XY | 46,XY | 46,XY | 46,XY | 46,XY | 46,XY | 46,XY |
| Age at presentation | 2m | 30y | 7y8m | 35y | 19y | 3y8m | Adult | Adult | Adult | Adult | 20y |
| Diagnosis | ETRS | ETRS | PGD | Previous gonadectomy | ETRS | PGD | pvDSD | pvDSD | pvDSD | pvDSD | Gonadal dysgenesis |
| External genitalia | Micropenis | Micropenis | Female | NA | Micropenis | Atypical | Partially virilized | Partially virilized | Partially virilized | Partially virilized | primary  amenorrhea |
| Internal genitalia | no Müllerian structures , Wolffian structures present | Wolffian  structures and Müllerian structures present | no Müllerian structures, Wolffian structures present | NA | no  Müllerian structures | Wolffian  structures and Müllerian structures present | No  Müllerian ducts | Vaginal septum/ uterine didelphys | No  Müllerian ducts | No  Müllerian ducts | No  Müllerian structures, Wolffian structures present |
| Gonad histology | No gonadal tissue | No gonadal tissue | Bilateral dysgenetic gonads | NA | No gonadal tissue | Bilateral dysgenetic | NA | NA | NA | NA | Homogeneous fibrous tissues associated with a rete testis on both sides |
| **LH, UI/L** |  |  |  |  |  |  |  |  |  |  |  |
| Value | 0.1 | 10 | 0.1 | NA | 23 | NA | NA | NA | NA | NA | 23.1 |
| Ref. Range |  |  |  |  | 2.4–13 |  |  |  |  |  | 2.4–13 |
| **FSH, UI/L** |  |  |  |  |  |  |  |  |  |  |  |
| Value | 0.4 | 40 | 4.9 | NA | 62 | NA |  |  | NA | NA | 61.5 |
| Ref. Range |  |  |  |  | ND–13.5 |  |  |  |  |  | ND–13.5 |
| **T, ng/mL** |  |  |  |  |  |  |  |  |  |  |  |
| Value | <10 | NA | <10 | NA | 21 | 25 | NA | NA | NA | NA | 0.29 |
| Ref. Range |  |  |  |  | 2.70–9.00 |  |  |  |  |  | 2.70–9.00 |
| **AMH, ng/mL** |  |  |  |  |  |  |  |  |  |  |  |
| Value | NA | NA | NA | NA | NA | NA | NA | NA | NA | NA | 0.29 |
| Ref. Range |  |  |  |  |  |  |  |  |  |  | 7.4–243 |
| Ancestry | Chinese American | Brazilian | Brazilian | Brazilian | Brazilian | Brazilian | European | European | European | European | European |
| *DHX37*pathogenic variant,zygosity and inheritance | c.923G>A p.Arg308Gln heterozygous de novo | c.923G>A p.Arg308Gln heterozygous unknown | c.923G>A p.Arg308Gln heterozygous de novo | c.911C>T p.Thr304Met heterozygous unknown | c.2020C>T p.Arg674Trp heterozygous unknown | c.2020C>T p.Arg674Trp heterozygous maternal | c.923G>A p.Arg308Gln heterozygous unknown | c.923G>A p.Arg308Gln heterozygous unknown | c.923G>A p.Arg308Gln heterozygous unknown | c.1430C>T p.Thr477Met heterozygous unknown | c.2021G>A p.Arg674Gln heterozygous  unknown |
| reference | da Silva et al.2019 | da Silva et al.2019 | da Silva et al.2019 | da Silva et al.2019 | da Silva et al.2019 | da Silva et al.2019 | Buonocore et al.2019 | Buonocore et al.2019 | Buonocore et al.2019 | Buonocore et al.2019 | McElreavey et al.2019 |

| **Variable** | | | Patient 23 | | Patient 24 | Patient 25 | | Patient 26 | | Patient 27 | | | Patient 28 | | Patient 29 | | Patient 30 | Patient 31 | |
| --- | --- | --- | --- | --- | --- | --- | --- | --- | --- | --- | --- | --- | --- | --- | --- | --- | --- | --- | --- |
| Sex of rearing | | | Female | | Female | Female | | Female | | Female | | | Female | | Female | | Female | Male | |
| Karyotype | | | 46,XY | | 46,XY | 46,XY | | 46,XY | | 46,XY | | | 46,XY | | 46,XY | | 46,XY | 46,XY | |
| Age at presentation | | | 3y | | 16y | 15y | | 15y6m | | Birth | | | Birth | | NA | | Birth | Birth | |
| Diagnosis | | | Gonadal dysgenesis | | Gonadal dysgenesis | Gonadal dysgenesis | | Gonadal dysgenesis | | Gonadal dysgenesis | | | 46,XY DSD | | 46,XY DSD | | Gonadal dysgenesis | TRS | |
| External genitalia | | | NA | | primary  amenorrhea | discrete  fusion of labia minora | | discrete  fusion of labia minora | | partial  fusion of labia minora | | | ambiguous  genitalia | | poorly  developed labia | | Ambiguous  genitalia | micropenis  and bilateral cryptorchidism | |
| Internal genitalia | | | Urogenital sinus | | No  Müllerian structures, Wolffian structures present | Fallopian  tube-like structures and epididymis on each side | | Vagina  present, absent uterus, Wolffian structures present (both sides) | | Vagina  16–17 mm long and 6–7 mm wide not opened, absent uterus; no gonads present | | | Vagina  present, absent uterus, Wolffian structures present | | Absent  uterus and vagina | | Vaginal  septum and uterus didelphys | No  Müllerian structures | |
| Gonad histology | | | Bilateral fibrous gonads, duct-like structures, fragments of uterine tube | | R: Small nodule of fibrous tissue; L: fibrous tissue with rare tubule- like structures | No gonadal tissue present | | Homogeneous fibrous tissue | | L: No gonad tissue, remnants of ductus deferens; R: no gonad tissue, remnants of epididymis tissue | | | NA | | NA | | NA | No gonadal tissue; remnants of epididymis, ductus deferens | |
| **LH, UI/L** | | |  | |  |  | |  | |  | | |  | |  | |  |  | |
| Value | | | 7.2 (4 years） | | 18 | 17 | | 20 | | 0.7 (1 w) ,3.3 (5 m) | | | NA | | NA | | NA | 0.6 (1 w) | |
| Ref. Range | | | Tanner 1: 0.03–0.55 | | 2.4–13 | 2.4–13 | | 2.4–13 | | 0.2–0.5 | | | NA | | NA | | NA | 0.2–0.5 | |
| **FSH, UI/L** | | |  | |  |  | |  | |  | | |  | |  | |  |  | |
| Value | | | 90.9 (4 years) | | 56 | 45 | | 50 | | 1.2 (1 w) ,54.4 (5 m) | | | NA | | NA | | NA | 10.7 (1 w) | |
| Ref. Range | | | 0.7–3.39 | | ND–13.5 | ND–13.5 | | ND–13.5 | | ND–2.0 | | | NA | | NA | | NA | ND–2.0 | |
| **T, ng/mL** | | |  | |  |  | |  | |  | | |  | |  | |  |  | |
| Value | | | <0.025 (4 y) | | <0.25 | <0.25 | | <0.25 | | 0.25 (1 w),<0.19 (5 m) | | | NA | | NA | | NA | 0.16 (1 w),2 (9 w) | |
| Ref. Range | | | 0.02–0.23 | | 2.70–9.00 | 2.70–9.00 | | 2.70–9.00 | | 0.1–0.5 0.02–0.2 | | | NA | | NA | | NA | 0.1–0.5 ,0.8–3.3 | |
| **AMH, ng/mL** | | |  | |  |  | |  | |  | | |  | |  | |  |  | |
| Value | | | 0.06 (4 y) | | NA | 0.65 | |  | | 0.01 (5 m) | | |  | |  | |  |  | |
| Ref. Range | | | 32.77-252.69 | |  | 7.4–243 | | 7.4–243 | | 105–270 | | |  | |  | |  |  | |
| Ancestry | | | European | | European | European | | European | | European | | | European | | European | | European | European | |
| DHX37pathogenic variant,zygosity and inheritance | | | c.2021G>A p.Arg674Gln  heterozygous de novo | | c.923G>A p.Arg308Gln heterozygous de novo | c.911C>T p.Thr304Met heterozygous maternal | | c.911C>T p.Thr304Met heterozygous de novo | | c.1001G>T p.Arg334Leu heterozygous de novo | | | c.923G>A p.Arg308Gln heterozygous unknown | | c.923G>A p.Arg308Gln heterozygous unknown | | c.923G>A p.Arg308Gln heterozygous unknown | c.1877C>T p.Ser626Leu heterozygous unknown | |
| reference | | | McElreavey et al.2019 | | McElreavey et al.2019 | McElreavey et al.2019 | | McElreavey et al.2019 | | McElreavey et al.2019 | | | McElreavey et al.2019 | | McElreavey et al.2019 | | McElreavey et al.2019 | McElreavey et al.2019 | |
| **Variable** | Patient 32 | | Patient 33 | | | Patient 34 | | Patient 35 | | Patient 36 | Patient 37 | | Patient 38 | | Patient 39 | | | Patient 40 | |
| Sex of rearing | Male | | Male | | | Male | | Male | | Male | Male | | Male | | Male | | | Female | |
| Karyotype | 46,XY | | 46,XY | | | 46,XY | | 46,XY | | 46,XY | 46,XY | | 46,XY | | 46,XY | | | 46,XY | |
| Age at presentation | Birth | | Birth | | | Birth | | 45d | | 20 m | Birth | | Birth | | 21 m | | | 14 y | |
| Diagnosis | TRS | | TRS | | | TRS | | TRS | | TRS | TRS | | TRS | | DSD | | | Suspected CGD | |
| External genitalia | micropenis, hypospadias; unilateral cryptorchidism (L); small palpable testis (R) | | severe  micropenis; cryptorchidism | | | bilateral  cryptorchidism | | Micropenis (<5 mm),posterior hypospadias,bilateral cryptorchidismnon-palpable gonads, hypoplastic labia | | Micropenis (5 mm),bilateral cryptorchidism,non-palpable gonads,fused pigmented labia,fused labia minora | Micropenis (12×9mm), non-palpable gonads,bilateral cryptorchidism | | Micropenis (8×5mm), midshaft  hypospadias, non_x005f palpable gonads, bilateral  cryptorchidism | | Micropenis (<5mm), bilateralcryptorchidism,  non-palpable gonads,poorly developed and fused labia | | | Female | |
| Internal genitalia | Vagina  present | | Absent vagina and uterus; bilateral epididymal-like structures | | | Absent gonads with vas deferens present (12 years) | | Müllerian ducts  present | | No  Müllerian ducts | No  Müllerian ducts | | No  Müllerian ducts | | No  Müllerian ducts | | | NA | |
| Gonad histology | NA | | L: Homogeneous fibrous tissue; R: no gonadal tissue | | | NA | | NA | | NA | NA | | NA | | NA | | | NA | |
| **LH, UI/L** |  | |  | | |  | |  | |  |  | |  | |  | | |  | |
| Value | 0.5 (28 d) | | <0.4 (10 d) | | | NA | | 0.20 (3 m) | | 11.38 (6 m) | <0.1 (2 d), 0.5 (1 m) | | 0.5 (1 m) | | NA | | | 45.2 | |
| Ref. Range | 0.2–0.5 | | 0.2–0.5 | | |  | | 1.1–25 (3 m) | | 1.1–25 (3 m) | <2 (2 d),0.9–3 (1 m) | | 0.9–3 (1 m) | |  | | | 2.4–13.0 | |
| **FSH, UI/L** |  | |  | | |  | |  | |  |  | |  | |  | | |  | |
| Value | 8.9 (28 d) | | 26.7 (5 y) | | | 100 (2 y) | | 8.88 (3 m) | | 86.76 (6 m) | <0.3 (2 d), 6.9 (2 d) | | 8.9 (1 m), 52.4 (3 y) | | NA | | | 76.7 | |
| Ref. Range | ND–2.0 | | ND–2.0 | | | 0.22–1.92 | | 1.5–11.8 (3 m) | | 1.5–11.8 (3 m) | <2 (2 d),<3 (1 mo) | | <3 (1 m), <2 (3 y) | |  | | | ND–13.5 | |
| **T, ng/mL** |  | |  | | |  | |  | |  |  | |  | |  | | |  | |
| Value | 0.1 (Basal and after HCG 1500 ui x3) | | 0.2 (10 d) | | | 0.7 (11 y) | | 0.13 (3 m) | | 0.03 (6 m) | 0.6 (2 d), <0.10 (1 m) | | <0.10(1 m) | | <0.2 (25 m) | | | NA | |
| Ref. Range | 0.2–0.5 | | 0.7–1.7 | | | (0.4–19) | | 2.2–10.5 (3 m) | | 2.2–10.5 (3 m) | 0.4–3 (2 d),1–3 (1 m) | | 1–3 (1 m) | |  | | |  | |
| **AMH, ng/mL** |  | |  | | |  | |  | |  |  | |  | |  | | |  | |
| Value |  | | 0 | | | <0.01 (11 y) | | <0.03 (3 m) | | 0.01 (6 m) | <0.1 (2 d), <0.2 (1 m) | | NA | | 38 (25 m) | | | NA | |
| Ref. Range |  | |  | | | 7.4–243 | |  | | 2.0–6.8 (6 m) | >28 (2 d),>51.8 (1 m) | |  | | 21–210 (25 m) | | |  | |
| Ancestry | European | | European | | | European | | Algerian | | Algerian | French | | French | | Algerian | | | Iranian | |
| *DHX37*pathogenic variant,zygosity and inheritance | c.1000C>T p.Arg334Trp heterozygous unknown | | c.923G>A p.Arg308Gln heterozygous de novo | | | c.3089G>A p.Gly1030Glu heterozygous unknown | | c.923G>A p.Arg308Gln heterozygous de novo | | c.923G>A p.Arg308Gln heterozygous maternal | c.923G>A p.Arg308Gln heterozygous unknown | | c.1000C>T p.Arg334Trp heterozygous unknown | | c.1460G>A p.Arg487His heterozygous paternal | | | c.1169G>A p.Arg390His heterozygous unknown | |
| reference | McElreavey et al.2019 | | McElreavey et al.2019 | | | McElreavey et al.2019 | | Zidoune et al.2021 | | Zidoune et al.2021 | Zidoune et al.2021 | | Zidoune et al.2021 | | Zidoune et al.2021 | | | Zidoune et al.2021 | |

| **Variable** | Patient 41 | Patient 42 | Patient 43 | Patient 44 | Patient 45 | Patient 46 | Patient 47 | Patient 48 | Patient 49 |
| --- | --- | --- | --- | --- | --- | --- | --- | --- | --- |
| Sex of rearing | Male | Male | Male | Female | Female | Male | Male | Female | NA |
| Karyotype | 46,XY | 46,XY | 46,XY | 46,XY | 46,XY | 46,XY | 46,XY | 46,XY | 46,XY |
| Age at presentation | Birth | 15 m | 4y5m | 13y6m | 15y | 10y | 1m | 17y | 0.5m |
| Diagnosis | TRS | DSD | TRS | GD | TRS | PGD | PGD | PGD | PGD |
| External genitalia | Micropenis (15×6mm),non-palpable gonads,bilateral cryptorchidism | Hypospadias (Scrotal) Penis Dysplasia Bilateral Cryptorchidism | Micropenis and bilateral cryptorchidism | Female with mild posterior labial fusion | Poorly developed labia, clitoral hypertrophy, absence of vaginal opening | Micropenis, hypoplastic scrotum with non-palpable testis, hypospadias | with a 1.0 cm phallus, normal male urethral opening without hypospadias, and both gonads palpable in the inguinal canal(Prader 5; EMS 8.0; EGS 8.5) | female external genitalia with clitoromegaly(3 cm) | ambiguous genitalia, with a 2.2 cm phallus length with chordee, a penoscrotal urethral opening |
| Internal genitalia | No  Müllerian ducts | NA | Müllerian ducts present No Wolffian derivatives | NA | No mullerian structures, no gonads present | No mullerian structures, no gonads present | No mullerian structures | no palpable gonads | both gonads palpable in the labioscrotal folds |
| Gonad histology | NA | NA | NA | NA | NA | Fallopian tube- and epididymis-like structures on both sides, remnants of the ductus deferens on the left side | NA | dysgenetic testes in both sides with the presence of Sertoli cells, but few Leydig and germ cells, with no ovarian tissue present | NA |
| **LH, UI/L** |  |  |  |  |  |  |  |  |  |
| Value | <0.1 (2 d), 25.4 (17 d) | NA | 1.06 | 41.37 | 19.32 | 1.15 | 2.7 | 13.9 | 7.5 |
| Ref. Range | <2 (2 d), 0.9–3 (17 d) |  | <0.2 | <22.04 | 0.57- 12.07 | 0.57- 12.07 | 0.6–3.5 | 0.6–8.5 | 0.6–3.5 |
| **FSH, UI/L** |  |  |  |  |  |  |  |  |  |
| Value | 0.5 (2 d),66.8 (17 d) | NA | 25.5 | 102.55 | 73.34 | 17.27 | 12.4 | 72.1 | 11.8 |
| Ref. Range | <2 (2 d),<3 (17 d) |  | <0.7 | 1.24–8.64 | 0.95- 11.95 | 0.95- 11.95 | 0.5–4.5 | 0.5–9.5 | 0.5–4.5 |
| **T, ng/mL** |  |  |  |  |  |  |  |  |  |
| Value | 0.70 (2 d),0.30 (17 d) | NA | <0.69 | 0.16 | 0.4 | 0.22 | 154 | 70 | 108 |
| Ref. Range | 0.4-3 (2d), 1–3 (17d) |  | <1.04 | <2.04 | 4.94- 32.01 | 4.94- 32.01 | 100–300 | 100–750 | 100-300 |
| **AMH, ng/mL** |  |  |  |  |  |  |  |  |  |
| Value | <0.3 (2 d), 0.4 (17 d) | NA | 0.43 | 0.01 | 0.02 | NA | NA | NA | NA |
| Ref. Range | >28(2d),>51.8 (17d) |  | 23.70–250.35 | 0.97–11.76 | 0.96- 13.34 | 0.96- 13.34 |  |  |  |
| Ancestry | French | Chinese | Chinese | Chinese | Chinese | Chinese | Brazilian | Brazilian | Brazilian |
| DHX37pathogenic variant,zygosity and inheritance | c.1430C>T p.Thr477Met homozygous unknown | c.923G>A p.Arg308Gln heterozygous de novo | c.1432G>A p.Gly478Arg heterozygous maternal | c.1879C>A  p.Leu627Phe heterozygous maternal | c.1223C>T  p.Ser408Leu heterozygous maternal | c.1223C>T  p.Ser408Leu heterozygous maternal | c.923G>A p.Arg308Gln heterozygous unknown | c.1399C>G p.Leu467Val heterozygous maternal | c.2995G>A p.Val999Met heterozygous Paternal |
| reference | Zidoune et al.2021 | Shaomei et al.2022 | Yang H.2023 | Yang H.2023 | Wan et al.2023 | Wan et al.2023 | de Oliveira et al.2023 | de Oliveira et al.2023 | de Oliveira et al.2023 |

| **Variable** | Patient 50 | Patient 51 | Patient 52 | Patient 53 | Patient 54 | Patient 55 | Patient 56 | Patient 57 | Patient 58 |
| --- | --- | --- | --- | --- | --- | --- | --- | --- | --- |
| Sex of rearing | NA | NA | NA | NA | NA | NA | Female | male | male |
| Karyotype | 46,XY | 46,XY | 46,XY | 46,XY | 46,XY | 46,XY | 46,XY | 46,XY | 46,XY |
| Age at presentation | 12y | NA | NA | NA | NA | 31y | 3y | 12.9y | 0.3y |
| Diagnosis | PGD | NA | NA | NA | NA | DSD | GD | DSD | DSD |
| External genitalia | The patient had a 4 cm phallus length, a distal penile urethral opening | NA | NA | NA | NA | NA | Inguinal hernia at 3 yr | NA | NA |
| Internal genitalia | No mullerian structures | NA | NA | NA | NA | No mullerian structures | Abdominal cavity | Bilateral  cryptorchidism | Inguinal |
| Gonad histology | NA | NA | NA | NA | NA | NA | fibrosis, structures similar to ducts, fragments of the uterine tube with sclerosis | NA | NA |
| **LH, UI/L** |  |  |  |  |  |  |  |  |  |
| Value | 2.8 | NA | NA | NA | NA | NA | NA | 3.3 | 0.1 |
| Ref. Range | 0.6–8.5 |  |  |  |  |  |  |  |  |
| **FSH, UI/L** |  |  |  |  |  |  |  |  |  |
| Value | 20.7 | NA | NA | NA | NA | 86.56 | NA | 7 | 2.5 |
| Ref. Range | 0.5–9.5 |  |  |  |  |  |  |  |  |
| **T, ng/mL** |  |  |  |  |  |  |  |  |  |
| Value | 20 | NA | NA | NA | NA | NA | NA | 619 | <12 |
| Ref. Range | 100–750 |  |  |  |  |  |  |  |  |
| **AMH, ng/mL** |  |  |  |  |  |  |  |  |  |
| Value | NA | NA | NA | NA | NA | NA | NA | NA | NA |
| Ref. Range |  |  |  |  |  |  |  |  |  |
| Ancestry | Brazilian | Chinese | Chinese | Chinese | Chinese | India | Ukrainian | Brazilian | Brazilian |
| *DHX37*pathogenic variant,zygosity and inheritance | c.2995G>A p.Val999Met heterozygous unknown | c.760A>G p.Ile254Val heterozygous unknown | c.923G>A p.Arg308Gln heterozygous unknown | c.923G>A p.Arg308Gln heterozygous unknown | c.1516G>A p.Asp506Asn heterozygous unknown | c.1877C>T p.Ser626Leu heterozygous de novo | c.2021G>A p.Arg674Gln heterozygous  unknown | c.2209G>A p.Ala737Thr heterozygous  unknown | c.1474G>C p.Ala492Pro heterozygous  unknown |
| reference | de Oliveira et al.2023 | Zhang et al.2023 | Zhang et al.2023 | Zhang et al.2023 | Zhang et al.2023 | Kulkarni V2023 | Globa et al.2022 | Gomes et al.2022 | Gomes et al.2022 |
